# Association and causal impact of TERT genetic variants on peripheral blood leukocyte telomere length and cerebral small vessel disease risk in a Chinese Han population: a mendelian randomization analysis

**DOI:** 10.1186/s13023-024-03316-5

**Published:** 2024-08-23

**Authors:** Ying Song, Jialiang Xu, Wanru Geng, Long Yin, Jialu Wang, JiuHan Zhao

**Affiliations:** 1 Department of Neurology, Affiliated Hospital of Inner Mongolia Minzu University, Tongliao, Inner Mongolia Autonomous Region, 028000 China; 2 Department of Cerebrovascular Disease Treatment Center, The People’s Hospital of Liaoning Province, Shenyang, Liaoning Province, 110002 China; 3 Department of Neurology, First Affiliated Hospital of China Medical University, Shenyang, Liaoning Province, 110001 China

**Keywords:** Cerebral small vessel diseases, TERT, Telomere length, Polymorphism, Mendelian randomization

## Abstract

**Background:**

Previous observational studies have highlighted potential relationships between the telomerase reverse transcriptase (TERT) gene, short leukocyte telomere length (LTL), and cerebrovascular disease. However, it remains to be established as to whether TERT gene variants are associated with an elevated risk of cerebral small vessel disease (CSVD), and whether there is a causal relationship between LTL and CSVD.

**Methods:**

Five TERT single nucleotide polymorphisms (SNPs) were analyzed in 307 CSVD patients and 320 healthy controls in whom LTL values were quantified. Allele models and four genetic models were used to explore the relationship between these SNP genotypes and CSVD risk. A Mendelian randomization analysis of CSVD risk was then performed using LTL-related SNPs and the polygenic risk score (PRS) constructed from these SNPs as genetic instrumental variables to predict the causal relationship between LTL and CSVD risk.

**Results:**

Model association analyses identified two SNPs that were significantly associated with CSVD risk. LTL was significantly correlated with age (*P* < 0.001), and the MR analysis revealed an association between short LTL and an elevated risk of CSVD. PRS-based genetic prediction of short LTLs was also significantly related to an elevated CSVD risk.

**Conclusion:**

Multiple genetic models and MR results indicate that TERT gene SNPs may be related to an elevated risk of CSVD, and that shorter LTL may be causally linked to such CSVD risk.

## Introduction

Cerebral small vessel disease (CSVD) refers to a common group of cerebrovascular diseases exhibiting varying imaging features [1]. Prior evidence suggests that CSVD is a complex polygenetic disease influenced by several risk factors, yet the precise genetic basis for CSVD risk remains to be fully elucidated [2, 3].

The telomerase reverse transcriptase functions to continuously extend or maintain the length of telomeres in response to activation by a telomerase RNA template. Components of the telomerase complex include telomerase-associated protein, telomerase reverse transcriptase (TERT), and telomerase RNA component. Of these, TERT is an essential regulator of telomerase activity [4, 5], and observational analyses suggest that TERT gene variants are related to ischemic stroke (IS) risk [6]. We thus hypothesized that TERT gene variants may also be associated with patient susceptibility to CSVD.

Leukocyte telomere length (LTL) has long been analyzed as an aging-related biomarker [7]. Several single nucleotide polymorphisms (SNPs) proximal to the TERT gene have been reported to be associated with LTL, but whether these TERT variants play any functional role in disease pathogenesis remains uncertain [8, 9]. While IS risk has been linked with LTL shortening, no similar evidence regarding a link between LTL and CSVD risk has been published to date. Traditional observational studies are limited in their ability to assess causal relationships between LTL and CSVD owing to bias stemming from the inability to fully control for confounding variables, reverse causality, minor exposure factors, and multiple testing. To address these issues, epidemiological research has increasingly employed Mendelian randomization (MR) studies in which select genetic mutations are treated as instrumental variables (IVs). While MR studies of the causal relationship between LTL and several diseases have been performed in recent years, there have not been any specific MR analyses of the interplay between LTL and CSVD risk [10, 11].

Here, an MR approach was used to explore the relationship between TERT gene variants, LTL, and CSVD by correcting for confounding factors and other biases. These efforts and a further exploration of the causal effect of LTL on CSVD incidence offer a new foundation for assessing CSVD risk.

## Materials and methods

### Study population

In total, 307 consecutive CVSD patients 45–75 years old that were admitted to the outpatient department and wards of the Liaoning Provincial People’s Hospital between October 2018 and August 2020 whose CVSD diagnosis was consistent with the 2021 Chinese Consensus for the Diagnosis and Treatment of Cerebral Small Vascular Diseases were enrolled in this study. In addition, 320 healthy controls were recruited. All participants were unrelated individuals of Han ethnicity. This study was carried out in accordance with the Helsinki Declaration and approved by the Ethics Committee of Liaoning Provincial People’s Hospital (No. (2021)HS007). Participants gave informed consent to participate in the study before taking part.

**Fig. 1:**
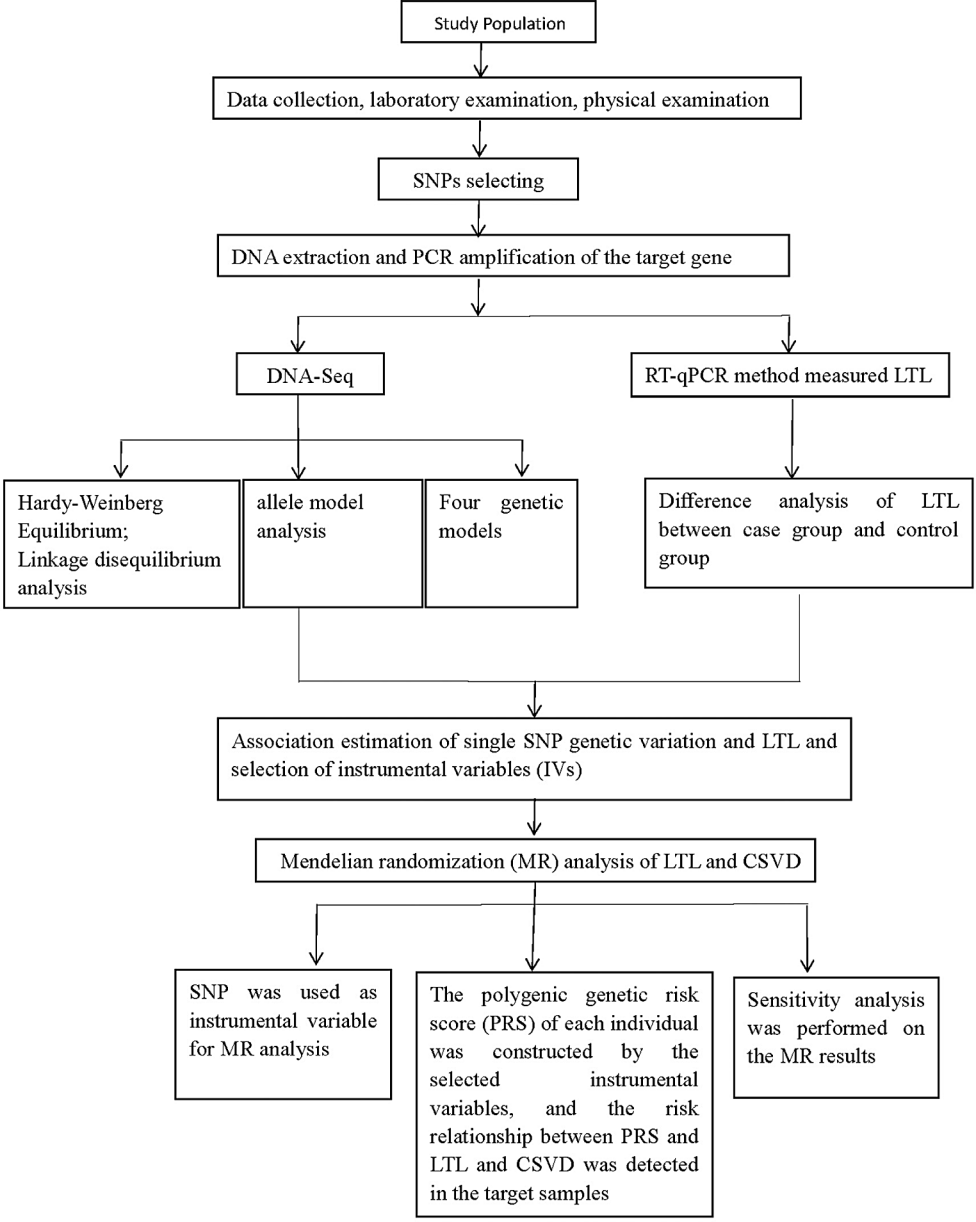
Study details

All subjects signed the informed consent form. The specific research process is illustrated in Fig. 1.

Inclusion criteria: (1) residents of Liaoning Province for ≥ 30 years; (2) 45–75 years old; (3) unrelated to other enrolled patients; (4) confirmed CSVD diagnosis as per the “China Consensus on Diagnosis and Treatment of Cerebral Small Vascular Diseases” (2021), with typical imaging findings and consistent acute or subacute clinical manifestations.

Exclusion criteria: (1) atherosclerotic thrombosis, or a history of transient ischemic attack (TIA) or intracranial hemorrhage; (2) cervical vascular ultrasound or head MRA results consistent with the moderate-to-severe occlusion or stenosis of vessels in the head and neck; (3) other neurological diseases including epilepsy, multiple sclerosis, hydrocephalus, or intracranial infectious diseases; (4) white matter lesions of known etiology, including neurosyphilis, carbon monoxide positioning, or degenerative disease; (5) history of serious heart diseases including coronary heart disease, atrial fibrillation, myocardial infarction within the past 6 months, or heart failure; (6) severe liver and kidney diseases or malignant tumors; (7) severe coagulation disorders or active bleeding.

### Data collection

Medical records or in-person questionnaires were used to collect data including age, sex, height, weight, systolic blood pressure (SBP), diastolic blood pressure, smoking history, alcohol consumption, and history of physical activity from all participants. Standard methods were used to analyze laboratory parameters including levels of fasting blood glucose, triglycerides, total cholesterol, high-density lipoprotein cholesterol, low-density lipoprotein cholesterol (LDL-C), and homocysteine (Hcy) in the clinical laboratory of Liaoning Provincial People’s Hospital. Both cases and controls underwent TERT genotyping and LTL measurements.

### SNP selection and genotyping

Five SNPs with a minor allele frequency ≥ 5% were selected based on the GWAS database (https://www.ebi.ac.uk/gwas) and published research: rs2853676, rs2242652, rs2075786, rs2736100, rs2736122. Fasting venous blood (3 mL) was collected from all subjects in EDTA-containing tubes. Shanghai Sangon Company extracted whole genomic DNA from these samples and synthesized appropriate SNP locus primers. DNA amplification was performed with the following settings: 95 °C for 5 min; 38 cycles of 94 °C for 30 s, 58 °C for 30 s, and 72 °C for 60 s; 72 °C for 10 min. Then, 1% agarose gel electrophoresis (10–20 min, 150 V, 100 mA) was used to separate 5 µl of amplified DNA per sample, followed by visualization with a UV gel imaging system. A3730XL sequencer (ABI Company, US) was then used to sequence these amplified samples for genotyping.

### LTL measurements

A qPCR approach was used to measure LTL using the following primers (5’-3’) specific for the human telomere and β-globin genes:

TEL (forward): GGTTTTTGAGGGTGAGGGTGAGGGTGAGGGTGAGGGT;

TEL (reverse): TCCCGACTATCCCTATCCCTATCCCTATCCCTATCCCTA;

HBG1 (forward): TCTGACACAACTGTGTTCACTAGC;

HBG1 (reverse): CACCAACTTCATCCACGTTCACC.

Cycle threshold (Ct) values were used to establish a standard curve as follows: T/S = [2 Ct (telomeres) / 2 Ct (single copy gene)] − 1 = Δ Ct, Δ Ct 1 T/S ratio for each sample, Δ Ct 2 T/S ratio for the reference gene; Δ Δ Ct = Δ Ct1 - Δ Ct 2, with the relative T/S ratio 2 ^− Δ Δ Ct^ representing relative LTL length.

### Statistical analysis

Data were analyzed with SPSS 26.0, R 4.0.3, and Stata 11. Normally distributed data are given as means ± standard deviation, whereas they were otherwise reported as medians. Qualitative data are reported as rates (%). Results were compared with independent sample t-tests of Fisher’s exact test, with Bonferroni correction for subsequent pairwise comparisons. The χ^2^ test was used to evaluate the Hardy-Weinberg equilibrium (HWE) of genotype distributions between the control and case groups. CSVD risk and associated 95% confidence interval (CI) values were estimated through a multivariate logistic regression analysis, with *P* < 0.05 as the significance threshold. Linkage disequilibrium analyses were performed with Haploview 4.2 (https://www.broadinstitute.org/Haploview), and SNP genetic correlation analysis modeling was performed using SNPStats (https://www.snpstats.net/start.htm).

A one-sample MR analysis was performed, with SNPs and telomere length-related PRS values being selected as IVs if they satisfied the following criteria: (1) selected SNPs were correlated with LTL; (2) the correlation between SNPs, LTL, and CSVD was not affected by confounding factors; (3) selected SNPs only affected CSVD via LTL and not via an alternative pathway. Direct correlation and causal relationships between LTL and CSVD were obtained with a two-stage least square regression model (2SLS) after eliminating confounders and reverse causality, after which the following were performed:

Step 1: Establish a G-X regression model to obtain the predicted value (P) for exposure factors.

Step 2: Construct a P-Y regression model, and derive the regression equation for the relationship between the predicted value of exposure factors P and outcome variable Y.

Quantitative estimates of the magnitude of the relationship between LTL and CSVD were made with a logistic regression model (θx = logOR), using *P* < 0.05 as the threshold for significance.

## Results

### Participant characteristics

This study enrolled 307 CSVD patients (156 female, 151 male; mean age 60.98 ± 8.165 years) and 320 healthy controls (148 female, 172 male; mean age 61.45 ± 8.280 years). CSVD patients exhibited significantly higher mean age, weight, body mass index (BMI) smoking status, blood pressure, blood glucose, LDL-C, and Hcy levels relative to controls (*P* < 0.05), whereas gender, height, triglyceride levels, cholesterol levels, physical activity, and alcohol consumption were comparable in these groups (Table 1).

**Table 1:** Distribution of general characteristics between the CSVD and control group

Variables	Case (*n* = 307)	Control (*n* = 320)	*P*
Age, year	60.99 ± 8.15	57.05 ± 5.89	1.26 × 10–11^*^
Gender	151(49.2%)	172(53.8%)	0.253
Male	151(49.2%)	172(53.8%)	
Female	156(50.8%)	148(46.2%)	
Height, cm	166.1 ± 8.30	166.96 ± 7.82	0.180
Weight, kg	72.09 ± 11.72	66.82 ± 10.45	4.64 × 10 − 9^*^
BMI, kg/m2(kg/m2)	26.01 ± 3.01	23.86 ± 2.75	1.77 × 10–19^*^
SBP, mmHg	148 ± 22.66	143.3 ± 21.61	0.008
DBP, mmHg	87.46 ± 12.9	83.06 ± 12.69	1.95 × 10 − 5^*^
Hcy, µmol/L	16.45 ± 5.88	13.04 ± 4.66	5.32 × 10–15^*^
TG, mmol/L	1.80 ± 1.04	1.73 ± 0.74	0.321
TC, mmol/L	5.06 ± 0.99	5.00 ± 0.76	0.388
LDL-C, mmol/L	2.95 ± 0.91	2.52 ± 0.96	1.87 × 10 − 8^*^
HDL-C, mmol/L	1.22 ± 0.44	1.02 ± 0.41	1.46 × 10 − 9^*^
Glu, mmol/L	6.28 ± 1.45	5.70 ± 1.87	1.92 × 10 − 5^*^
Smoking	95(30.9%)	68(21.2%)	0.006^*^
	212(69.1%)	252(78.8%)	
Drinking	83(27.0%)	90(28.1%)	0.760
	224(73.0%)	230(71.9%)	
Movement	73(23.8%)	83(25.9%)	0.532
	234(76.2%)	237(74.1%)	

*P < 0.05 indicates statistical significance. CSVD: cerebral small vessel disease disease;

### Linkage disequilibrium analysis

**Fig. 2:**
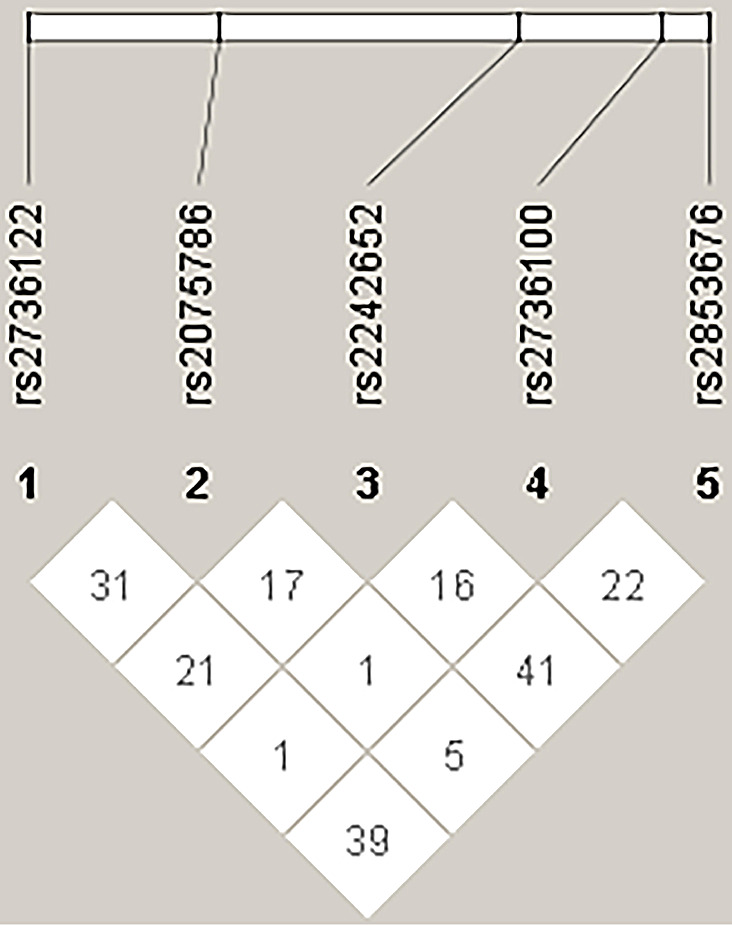
Linkage disequilibrium analysis

Linkage disequilibrium refers to the non-random association of different alleles in a population. Linkage disequilibrium analyses of these five SNPs did not reveal the formation of a haplotype block, and there was no evidence of strong linkage disequilibrium (Fig. 2).

### Relationship between SNPs and CSVD susceptibility

The relationship between individual SNPs and CSVD risk was next assessed. Using an allele model, the frequencies of the TERT rs2853676 and rs2075786 alleles differed significantly between CSVD patients and controls (OR = 1.43, 95%CI: 1.09–1.89, *P* = 0.001, and OR = 1.37, 95%CI: 1.09–1.73, *P* = 0.006 for the rs2853676 and rs2075786 allele, respectively). None of the analyzed SNPs deviated from the HWE in the control group (Table 2).

**Table 2:** The association between each SNP and CSVD risk predicted by allele gene model

SNP-ID	Alelle (A/B)	Case		Control		Case		Control		HWE-*P*	OR (95% CI)	X^2^	*P*
		A	B	A	B	Major	MAF	Major	MAF				
rs2853676	T/C	142	472	111	529	0.77	0.23	0.83	0.17	0.180	1.43 (1.09–1.89)	6.508	0.011^*^
rs2242652	A/G	116	498	104	536	0.81	0.19	0.84	0.16	0.840	1.20 (0.90–1.61)	1.513	0.219
rs2075786	A/G	257	357	220	420	0.58	0.42	0.66	0.34	0.390	1.37 (1.09–1.73)	7.442	0.006^*^
rs2736100	C/A	239	375	283	357	0.61	0.39	0.56	0.44	0.260	0.80 (0.64–1.01)	3.614	0.057
rs2736122	A/G	46	568	56	584	0.93	0.07	0.91	0.09	0.490	0.84 (0.56–1.27)	0.664	0.415

SNP: single nucleotide polymorphism; Major: maximum allele frequency; MAF: minimum allele frequency; HWE: Hardy Weinberg Equilibrium; OR: odds ratio; CI: confidence interval. *P < 0.05 indicates that the difference is statistically significant

The relationship between TERT gene polymorphisms and CSVD risk was further assessed using various genetic models (Table 3). Elevated CSVD risk was related to the rs2853676 C/T genotype under the codominant model (OR = 1.65, 95% CI: 1.11–2.43; *P* = 0.018), and the C/T and T/T genotypes under the dominant model (OR = 1.69, 95% CI: 1.17 to 2.46; *P* = 0.005) and log-additive model (OR = 1.53, 95% CI: 1.13–2.08; *P* = 0.006). Similarly, higher CSVD risk was related to the rs2075786 A/A genotype under the codominant model (OR = 2.01, 95% CI: 1.15–3.52; *P* = 0.021), and to the A/G and A/A genotypes under the dominant model (OR = 1.61, 95%CI: 1.12–2.33; *P* = 0.010) and log-additive model (OR = 1.44, 95% CI: 1.11–1.88; *P* = 0.006). For the rs2853676 SNP, higher CSVD risk was associated with the C/T genotype under the overdominant model (OR = 1.57, 95% CI: 1.07–2.31; *P* = 0.022), as was the rs2075786 A/G genotype under the co-dominant model (OR = 1.52, 95% CI: 1.03–2.23; *P* = 0.021) and rs2736122 with the A/G genotype under the overdominance model (OR = 0.59, 95% CI: 0.36–0.98; *P* = 0.042) after strictly adjusting for age, sex, BMI, smoking history, and alcohol consumption history.

**Table 3:** The association between each SNP and CSVD risk predicted by four genetic models

SNP-ID	Model	Genotype	control	case	Without adjustment				adjusted by Age + Gender + BMI + Smoking + Drinking			
					OR (95% CI)	*P*-value	AIC	BIC	OR (95% CI)	*P*-value	AIC	BIC
rs2853676	Codominant	C/C	222 (69.4%)	185 (60.3%)	1	0.046*	868.8	882.1	1	0.018*	744	779.5
		C/T	85 (26.6%)	102 (33.2%)	1.44 (1.02–2.04)*				1.65 (1.11–2.43)*			
		T/T	13 (4.1%)	20 (6.5%)	1.85 (0.89–3.81)				2.01 (0.88–4.58)			
	Dominant	C/C	222 (69.4%)	185 (60.3%)	1	0.017*	867.2	876.1	1	0.0052*	742.2	773.3
		C/T-T/T	98 (30.6%)	122 (39.7%)	1.49 (1.07–2.08)*				1.69 (1.17–2.46)*			
	Recessive	C/C-C/T	307 (95.9%)	287 (93.5%)	1	0.170	871	879.9	1	0.180	748.2	779.3
		T/T	13 (4.1%)	20 (6.5%)	1.65 (0.80–3.37)				1.73 (0.77–3.91)			
	Overdominant	C/C-T/T	235 (73.4%)	205 (66.8%)	1	0.068	869.6	878.5	1	0.022*	744.8	775.8
		C/T	85 (26.6%)	102 (33.2%)	1.38 (0.98–1.94)				1.57 (1.07–2.31)*			
	Log-additive	---	---	---	1.40 (1.07–1.83)*	0.014*	866.9	875.7	1.53 (1.13–2.08)*	0.006*	742.3	773.4
rs2242652	Codominant	G/G	225 (70.3%)	204 (66.5%)	1	0.450	873.4	886.7	1	0.250	749.2	784.7
		A/G	86 (26.9%)	90 (29.3%)	1.15 (0.81–1.64)				1.27 (0.86–1.89)			
		A/A	9 (2.8%)	13 (4.2%)	1.59 (0.67–3.81)				1.90 (0.71–5.13)			
	Dominant	G/G	225 (70.3%)	204 (66.5%)	1	0.300	871.9	880.7	1	0.140	747.8	778.9
		A/G-A/A	95 (29.7%)	103 (33.5%)	1.20 (0.85–1.68)				1.33 (0.91–1.94)			
	Recessive	G/G-A/G	311 (97.2%)	294 (95.8%)	1	0.330	872	880.9	1	0.250	748.7	779.7
		A/A	9 (2.8%)	13 (4.2%)	1.53 (0.64–3.63)				1.78 (0.66–4.75)			
	Overdominant	G/G-A/A	234 (73.1%)	217 (70.7%)	1	0.500	872.5	881.4	1	0.290	748.9	780
		A/G	86 (26.9%)	90 (29.3%)	1.13 (0.80–1.60)				1.24 (0.84–1.83)			
	Log-additive	---	---	---	1.19 (0.90–1.59)	0.230	871.5	880.3	1.31 (0.95–1.82)	0.100	747.3	778.4
rs2075786	Codominant	G/G	134 (41.9%)	100 (32.6%)	1	0.020*	867.1	880.4	1	0.021*	744.3	779.8
		A/G	152 (47.5%)	157 (51.1%)	1.38 (0.98–1.95)				1.52 (1.03–2.23)*			
		A/A	34 (10.6%)	50 (16.3%)	1.97 (1.19–3.27)*				2.01 (1.15–3.52)*			
	Dominant	G/G	134 (41.9%)	100 (32.6%)	1	0.016*	867.1	876	1	0.010*	743.3	774.4
		A/G-A/A	186 (58.1%)	207 (67.4%)	1.49 (1.08–2.07)*				1.61 (1.12–2.33)*			
	Recessive	G/G-A/G	286 (89.4%)	257 (83.7%)	1	0.037*	868.6	877.5	1	0.078	746.9	778
		A/A	34 (10.6%)	50 (16.3%)	1.64 (1.03–2.61)*				1.58 (0.95–2.65)			
	Overdominant	G/G-A/A	168 (52.5%)	150 (48.9%)	1	0.360	872.1	881	1	0.200	748.4	779.4
		A/G	152 (47.5%)	157 (51.1%)	1.16 (0.85–1.58)				1.26 (0.89–1.78)			
	Log-additive	---	---	---	1.40 (1.10–1.77)*	0.005*	865.1	874	1.44 (1.11–1.88)*	0.006*	742.4	773.5
rs2736100	Codominant	A/A	94 (29.4%)	107 (34.9%)	1	0.120	870.8	884.1	1	0.280	749.4	784.9
		A/C	169 (52.8%)	161 (52.4%)	0.84 (0.59–1.19)				0.93 (0.63–1.38)			
		C/C	57 (17.8%)	39 (12.7%)	0.60 (0.37–0.98)				0.65 (0.37–1.12)			
	Dominant	A/A	94 (29.4%)	107 (34.9%)	1	0.140	870.8	879.7	1	0.430	749.4	780.5
		A/C-C/C	226 (70.6%)	200 (65.2%)	0.78 (0.56–1.09)				0.86 (0.59–1.25)			
	Recessive	A/A-A/C	263 (82.2%)	268 (87.3%)	1	0.075	869.8	878.6	1	0.120	747.5	778.6
		C/C	57 (17.8%)	39 (12.7%)	0.67 (0.43–1.04)				0.67 (0.41–1.11)			
	Overdominant	A/A-C/C	151 (47.2%)	146 (47.6%)	1	0.930	872.9	881.8	1	0.690	749.8	780.9
		A/C	169 (52.8%)	161 (52.4%)	0.99 (0.72–1.35)				1.07 (0.76–1.52)			
	Log-additive	---	---	---	0.79 (0.62-1.00)*	0.047*	869	877.9	0.83 (0.64–1.08)	0.160	748.1	779.1
rs2736122	Codominant	G/G	265 (82.8%)	265 (86.3%)	1	0.110	870.5	883.8	1	0.081	747	782.5
		A/G	54 (16.9%)	38 (12.4%)	0.70 (0.45–1.10)				0.60 (0.36–0.99)			
		A/A	1 (0.3%)	4 (1.3%)	4.00 (0.44–35.99)				2.94 (0.27–32.42)			
	Dominant	G/G	265 (82.8%)	265 (86.3%)	1	0.220	871.5	880.3	1	0.077	746.9	777.9
		A/G-A/A	55 (17.2%)	42 (13.7%)	0.76 (0.49–1.18)				0.64 (0.39–1.05)			
	Recessive	G/G-A/G	319 (99.7%)	303 (98.7%)	1	0.150	870.9	879.7	1	0.320	749	780.1
		A/A	1 (0.3%)	4 (1.3%)	4.21 (0.47–37.85)				3.13 (0.28–34.39)			
	Overdominant	G/G-A/A	266 (83.1%)	269 (87.6%)	1	0.110	870.4	879.3	1	0.042*	745.8	776.9
		A/G	54 (16.9%)	38 (12.4%)	0.70 (0.44–1.09)				0.59 (0.36–0.98)*			
	Log-additive	---	---	---	0.85 (0.57–1.27)	0.420	872.3	881.2	0.71 (0.45–1.13)	0.150	747.9	779

The association between each SNP and CSVD risk by four genetic gene model; AIC: Akaike’s Information criterion; BIC: Bayesian Information criterion.*p-value < 0.05 indicates statistical significance

### Analyses of LTL in cases and control

**Fig. 3:**
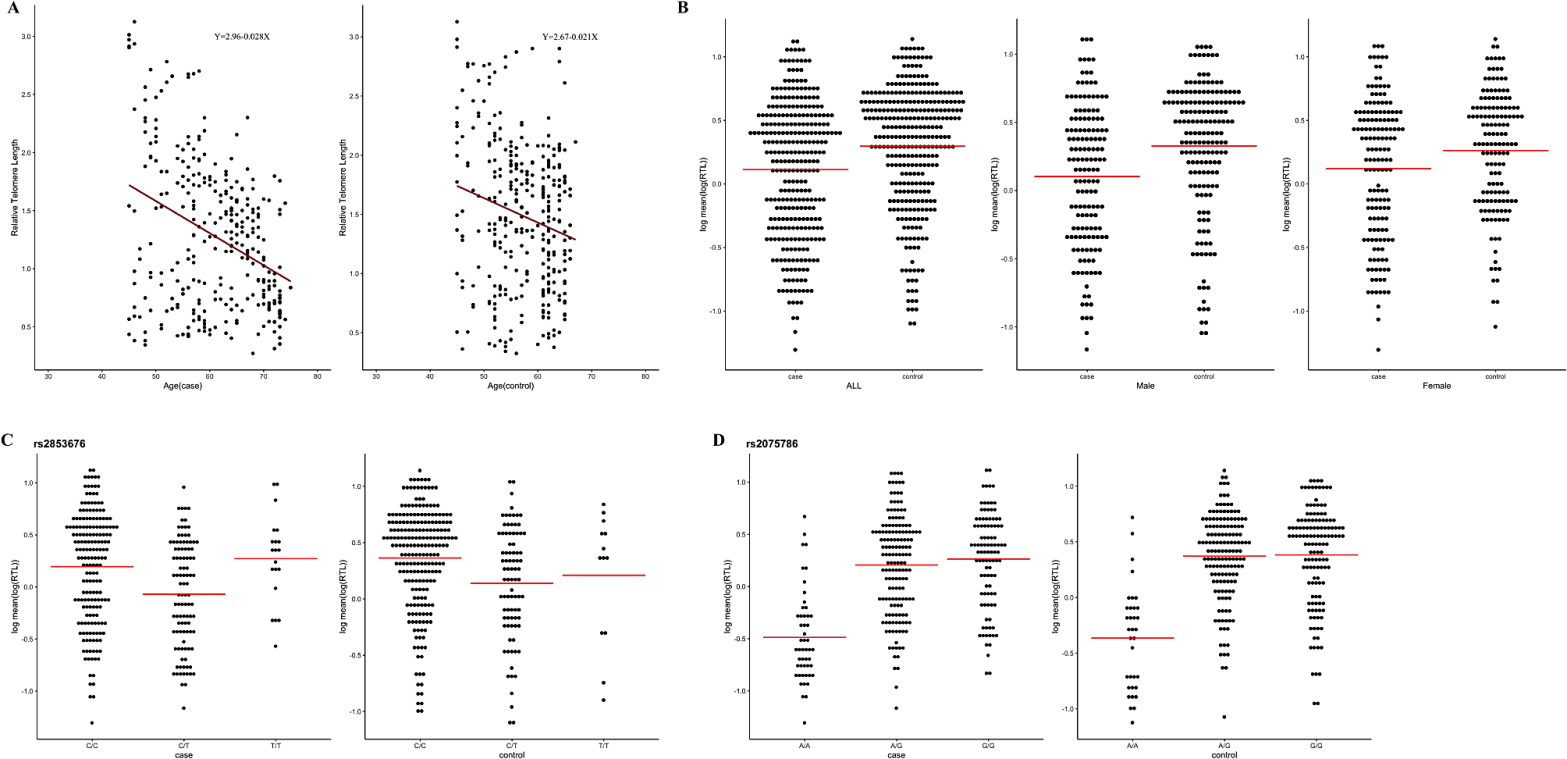
LTL analyses in cases and controls

LTL was negatively correlated with age in both the cases and controls (*P* < 0.001, Fig. 3A), with significantly different respective LTL values of 1.278 ± 0.634 and 1.492 ± 0.618 in these two groups (*P* < 0.001, Fig. 3B). No significant difference between the average LTL of males (1.405 ± 0.631; *n* = 323) and females (1.368 ± 0.638; *n* = 304) was observed (*P* = 0.463). However, CSVD patients exhibited a shorter LTL than controls among both males and females (males: 1.258 ± 0.621 vs. 1.534 ± 0.641, *P* < 0.001; females: 1.297 ± 0.649 vs. 1.442 ± 0.62, *P* = 0.048, Fig. 3B). Given the observed relationships between the rs2853786 and rs2075676 alleles in the TERT gene and CSVD risk, LTL was next compared for different genotypes in both the case and control populations. The respective LTL values for the rs2853786 C/C, C/T and T/T genotypes in the case group were 1.385 ± 0.669, 1.054 ± 0.510, and 1.427 ± 0.595 (*P* < 0.001), while those in controls were 1.577 ± 0.614, 1.283 ± 0.580, and 1.404 ± 0.632, respectively (*P* = 0.001, Fig. 3C). The LTL values associated with the rs2075786 A/A, A/G, and G/G genotypes among cases were 0.680 ± 0.357, 1.370 ± 0.620, and 1.433 ± 0.597, respectively (*P* < 0.001), while those for controls were 0.773 ± 0.394, 1.554 ± 0.550, and 1.603 ± 0.620, respectively (*P* < 0.001, Fig. 3D).

### Estimates of the relationship between individual SNPs and LTL, and instrumental variable selection

**Fig. 4:**
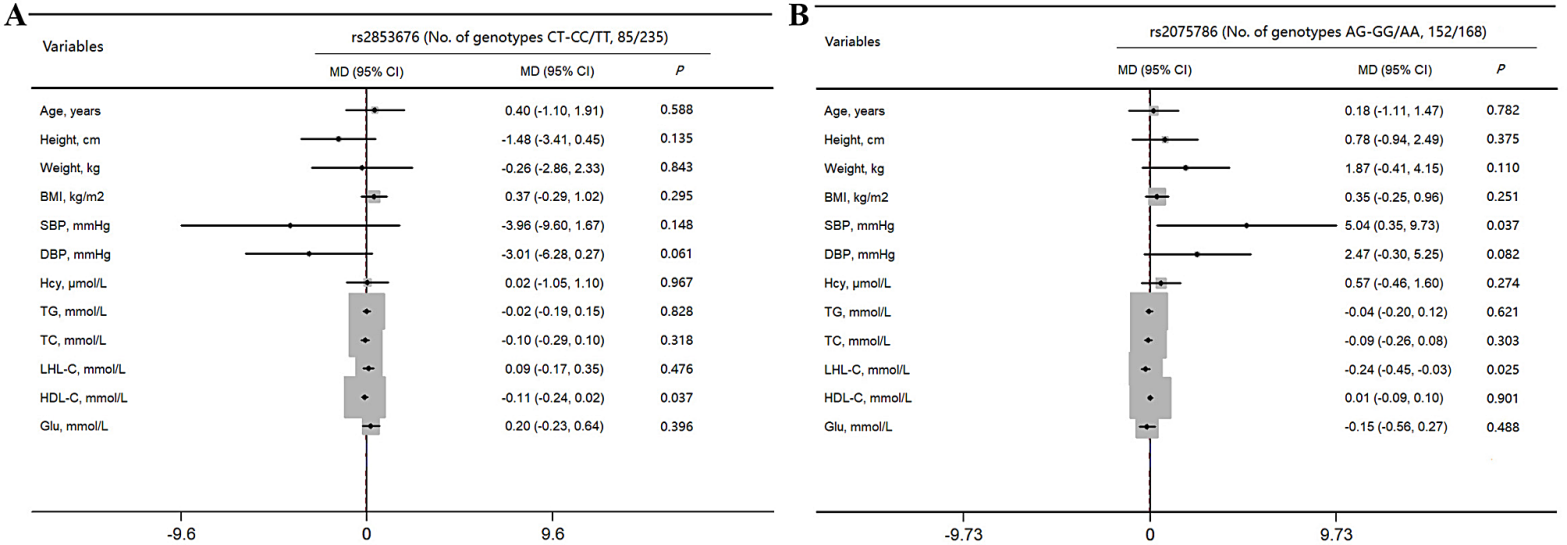
Correlations between SNP genotypes and potential confounding factors

Correlations between individual SNPs, CSVD, and LTL are summarized in Table 4. Following Bonferroni adjustment for age, sex, BMI, smoking history, and alcohol consumption history, three SNPs were significantly correlated with LTL (rs2853676, *P* = 1.09 × 10^− 5^; rs2242652, *P* = 1.38 × 10^− 4^; rs2075786, *P* = 7.12 × 10 ^− 21^). These results, together with the genetic model analyses performed above, led to the selection of rs2853676 and rs2075786 for further analyses examining associations between genotype and potential confounding factors (Fig. 4A-B). These analyses revealed no correlation between genotype and age, height, weight BMI, blood lipids, Hcy, or other analyzed risk factors (*P* > 0.05), although SBP did differ among rs2075786 genotypes (*P* = 0.037). This may be due to the limited sample size, which precluded the simultaneous satisfaction of multiple independent hypotheses. Accordingly, rs2853676, rs2242652, and rs2075786 were selected as IVs for a subsequent MR analysis.

**Table 4:** Correlation analysis of single SNPs in CSVD and LTL

SNP	effect_allele	other_allele	Beta	SE	*P*
rs2853676	T	C	-0.179292	0.04043	1.09 × 10^− 5*^
rs2242652	A	G	-0.16781	0.043753	1.38 × 10^− 4*^
rs2075786	A	G	-0.315562	0.033672	7.12 × 10^− 21*^
rs2736100	C	A	0.060489	0.036192	0.0952
rs2736122	A	G	-0.103726	0.061953	0.0946

*p-value < 0.05 indicates statistical significance

### Mendelian randomization analysis of the link between LTL and CSVD

**Fig. 5:**
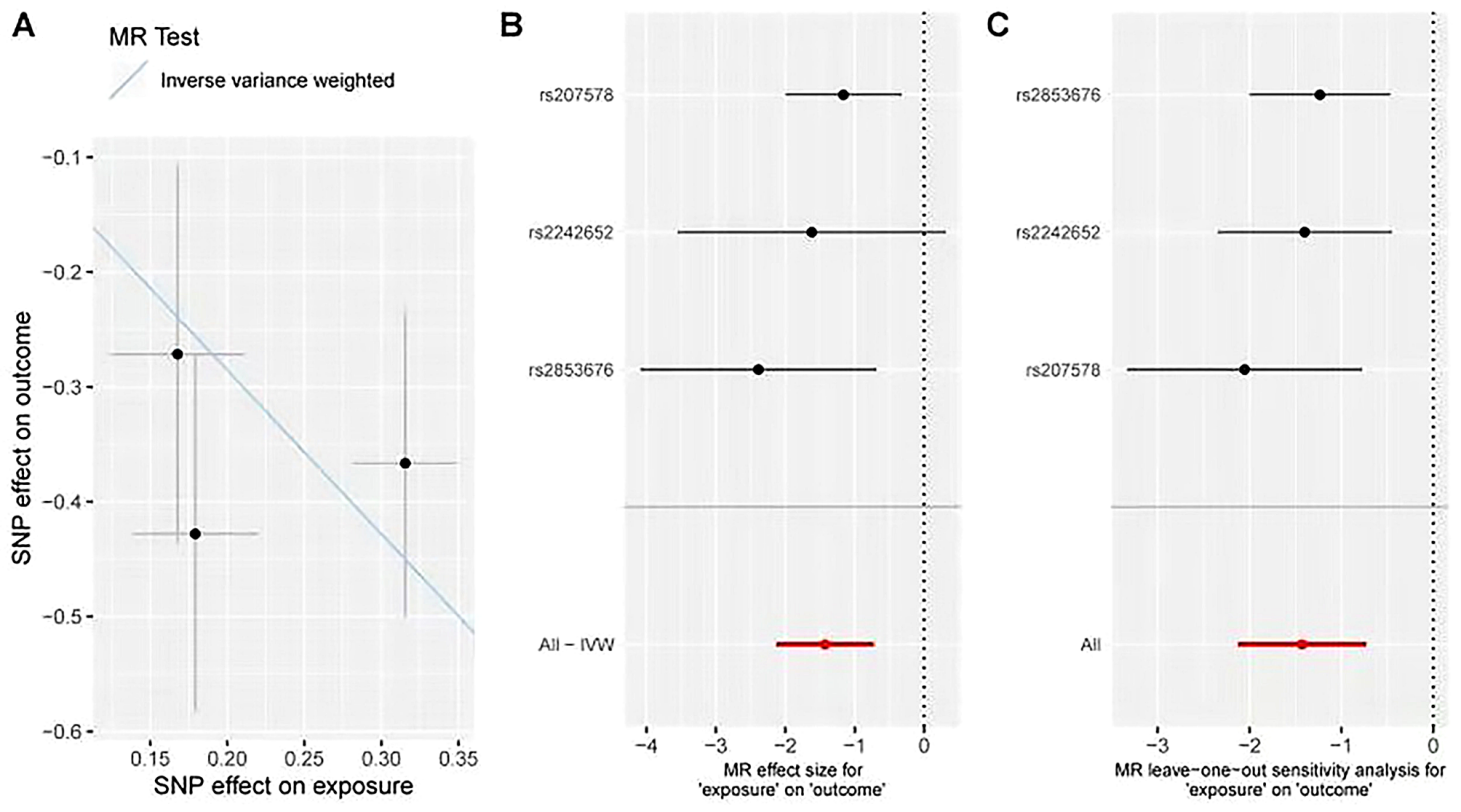
A: Scatterplot of the association of SNPs with LTL and CSVD risk; Each point in the scatter plot represents an instrumental variable. The vertical and horizontal black lines show the 95% CI for each SNP, with the horizontal axis illustrating the effect of the SNP on the exposure (telomere length), and the vertical axis highlighting the effect of the SNP on outcome (cerebrovascular disease), while the solid blue line shows the MR fitting results. B: Forest diagram of the risk relationship between LTL and CSVD, showing the odds ratio (OR), with the horizontal line representing the 95%CI of the risk of CSVD occurring in LTL-associated SNPs. The red line illustrates that shortening of LTLs increases the risk of CSVD. C: The sensitivity analysis of MR results was carried out by removing SNPs one by one

The causal effects of LTL on CSVD were assessed using a one-sample MR approach using a 2SLS. A PRS for telomere length was constructed from rs2853676, rs2242652, and rs2075786, and this PRS as well as the three individual SNPs were selected as IVs. Following Bonferroni correction, LTL values for different rs2853676 and rs2075786 genotypes remained strongly correlated with CSVD (Table 5). LTL was also significantly negatively correlated with the PRS (Beta = 0.991, *P* < 2 × 10^− 16^), while the PRS was significantly correlated with CSVD (Beta= -1.207, *P* = 1.84 × 10^− 4^). No significant confounding relationship was detected when exploring associations between the PRS and CSVD-associated risk factors, although LDL-C levels were related to the PRS (Beta = 0.419, *P* = 0.005, Table 6). Even so, following Bonferroni adjustment for LDL-C, a significant association between LTL and PRS remained evident (Beta =-1.066, *P* = 0.001). These results suggested that short LTL may have a causal impact on CSVD incidence. Leave-one-out sensitivity testing was used to confirm the accuracy of these results, with correlation analyses revealing that a significant correlation between IVs and CSVD remained present under these conditions, supporting the causal link between LTL and CSVD (Table 5; Fig. 5C).

**Table 5:** Mendelian randomization analysis of LTL and CSVD

SNP(IV)		2SLS			leave-one-out sensitivity test			
	Beta	SE	OR (95% CI)	*P*	Beta	SE	OR (95% CI)	*P*
rs2853676	-1.161	0.425	0.31(0.14–0.72)	6.35 × 10^− 3 *^	-2.052	0.652	0.13(0.04–0.46)	1.65 × 10^− 3 *^
rs2242652	-1.618	0.988	0.20(0.03–1.37)	0.101	-1.399	0.484	0.25(0.10–0.64)	3.88 × 10^− 3 *^
rs2075786	-2.387	0.868	0.09(0.02–0.50)	5.96 × 10^− 3 *^	-1.233	0.391	0.29(0.13–0.63)	1.61 × 10^− 3 *^
All SNPs	-1.427	0.356	0.24(0.12–0.48)	6.19 × 10^− 5 *^	-1.427	0.356	0.24(0.12–0.48)	6.19 × 10^− 5 *^

2SLS: least square method; *P < 0.05 indicates that the difference is statistically significant

**Table 6:** Association of the PRS with confounding factors

Confounder	Beta	SE	*P*
Age, year	-0.5482	1.1537	0.635
Gender	-0.02595	0.07849	0.741
Height, cm	0.8893	1.2649	0.482
Weight, kg	0.91	1.787	0.611
SBP, mmHg	-1.564	3.49	0.654
DBP, mmHg	-0.4568	2.0363	0.823
Hcy, µmol/L	1.211	0.8707	0.165
TG, mmol/L	0.2273	0.14096	0.107
TC, mmol/L	0.14484	0.13834	0.296
LDL-C, mmol/L	-0.41871	0.14961	0.00529
HDL-C, mmol/L	-0.03075	0.06858	0.654
Glu, mmol/L	-0.2722	0.2669	0.308
Smoking	-0.0429	0.06887	0.534
Drinking	-0.01064	0.0702	0.88
Movement	0.09711	0.06779	0.153

## Discussion

The present case-control study was constructed as the first analysis of the associations between SNPs in the TERT gene, LTL, and the risk of CSVD. LTL-associated SNPs were used as IVs for a one-sample MR analysis supplemented by the PRS method to fully explore potential causal relationships between LTL and CSVD. Five SNPs exhibiting a MAF greater than 5% were selected to ensure that these analyses were sufficiently powered. In the Chinese Han population, these analyses revealed a significant relationship between TERT gene variants and CSVD susceptibility, supporting a causal association between LTL and CSVD. These findings support the hypothesis that the TERT gene may contribute to elevated CSVD risk through LTL shortening.

Major pathogenic factors involved in the development of CSVD are thought to include inflammation, poor blood perfusion, blood-brain barrier impairment, and genetic susceptibility [12, 13]. In line with prior reports, CSVD risk was herein found to be associated with age, BMI, hypertension, hyperglycemia, dyslipidemia, smoking, and Hcy levels [12, 13]. Males have also been suggested to be at a higher risk of CSVD than females, potentially owing to sex-related differences in exposure to risk factors including smoking and drinking. The present results do not support a role for sex as a CSVD-related risk factor, although the limited study sample size limits further analyses of this relationship. In our study, we performed a correlation analysis of confounding risk factors associated with PRS and CSVD, the results showed that only LDL was associated with constructed PRS (Beta = 0.419, *P* = 0.005). After Bonferroni correction for LDL, we found that LTL and PRS were still statistically significant (Beta = -1.066, *P* = 0.001). Therefore, although there were differences in baseline data between the case and control groups, genetic factors were significantly associated with the development of LTL and CSVD.

TERT expression plays a key role in regulating telomerase activity [14]. Few studies to date, however, have assessed correlations between TERT polymorphisms and cerebrovascular diseases, and studies that have been completed have yielded inconsistent results among populations of different ethnicities. The ARIC study of 15,792 IS patients revealed an association between TERT gene polymorphisms and IS risk among African Americans, but found that this relationship was weaker when the regression model incorporated hypertension, diabetes, BMI, and smoking history in the regression model. Among Caucasians, no such link between TERT gene polymorphisms and IS risk has been observed [15]. In the population-based GWAS study by Wei et al. [16] the TERT rs2736100 SNP was reportedly significantly associated with LTL, while another report indicated that the TERT C and G alleles of the rs2853691 and rs33954691 genotypes were associated with shorter LTL. When studying TERT polymorphisms in the Chinese population, Zhang et al. [6]. found that the TERT rs224652 G/A (superdominant model) and A/A (dominant model) genotypes were associated with elevated IS risk. In our initial genetic analyses, TERT rs2242652 genotypes were not significantly correlated with CSVD but were significantly correlated with LTL (Beta=-0.168, Se = 0.044, *P* = 1.38 × 10^− 4^). In a study of African Americans, Bresler et al. [15]. found a significant association between the additive model of rs2853668 and ischemic stroke (HRR = 1.17, *p* = 0.05, 95% CI = 1.00–1.38).Han et al. [17] showed that the T/G and G/G genotypes of rs2736100 and the G/A and A/A genotypes of rs2853676 were associated with stroke risk (*P* < 0.05).This study was the first to assess the link between TERT SNPs and CSVD risk, ultimately revealing that rs2853676 and rs2075786 were significantly associated with CSVD even following Bonferroni correction. The rs2853676 C/T (co-dominant model) and C/T and T/T (dominant model and logarithmic additive model) genotypes were significantly associated with CSVD risk. Moreover, the rs2075786 A/A (co-dominant model) and A/G and A/A (dominant model and logarithmic additive model) genotypes were associated with elevated CSVD risk. Additionally, the rs2853676 C/T genotype (overdominant model), the rs2075786 A/G genotype (co-dominant model), and the rs2736122 A/G genotype (overdominant model) were significantly related to CSVD risk following strict adjustment for age, sex, BMI, smoking history, and alcohol consumption history. The level of significance declined following the correction of the logarithmic additive model for rs2736100, potentially indicating that rs2736100 may be correlated with the risk of CSVD but that further large-scale prospective analyses will be necessary to explore this possibility.

While prior evidence supports a correlative relationship between LTL and cerebrovascular disease risk, large-scale multi-center studies are lacking, and reported results have not been consistent. Gao et al. [18]. observed a significant correlation between LTL and age in a Chinese Han population (*P* < 0.001), and found shorter LTL to be associated with a higher risk of IS (OR = 8.44, 95%CI: 5.42–13.14, *P* < 0.001). This correlation between LTL shortening and IS risk was also supported by a meta-analysis of 11 studies [19]. When comparing stroke patients and controls (*n* = 1309 each), Ding et al. [20]. similarly observed a higher risk of IS among individuals with the shortest LTL relative to those with the longest LTL (OR = 2.12, 95%CI: 1.62–2.77). In contrast, Luo et al. [21]. reported detecting significantly longer LTL values in there is case group relative to their control and high-risk groups, whereas high-risk patients exhibited significantly shorter LTL as compared to controls, suggesting that the correlative relationship between LTL and IS risk may be U-shaped. Moreover, one 29-year Danish cohort study found that LTL was unrelated to IS incidence [22], and a 10-year prospective analysis conducted in the USA observed a positive correlation between shorter telomere length and stroke risk among individuals 65–73 years of age but not in individuals > 73 years old [23]. In the present case-control study, LTL-associated SNPs and PRSs constructed from these SNPs were used as instrumental variables in a one-sample MR analysis exploring the predicted association between LTL and CSVD risk. The results indicated that shorter LTL was associated with an elevated risk of CSVD. These findings align well well two recent GWAS data-based MR studies surveying a range of diseases that identified a possible causal relationship between short LTL and small vessel stroke (OR = 0.72, 95% CI: 0.54–0.97, *P* = 0.028) [24, 25]. Our results further suggest that LTL may be related to small cerebral vascular lesions.

In traditional observational studies, disease or treatment-related processes have the potential to contribute to LTL shortening, and both LTL and disease risk may be influenced by environmental variables. Under these conditions, establishing causality is challenging. This study exhibits several advantages including the use of a one-sample MR method and supporting sensitivity analyses of possible pluripotency effects, thereby mitigating potential bias stemming from the inability to fully control for confounding factors, reverse causality, minor exposure factors, and multiple testing. This is also the first MR study to explore the causal relationship between LTL and CSVD, as this association was not examined in prior analyses of the interplay between LTL and cerebrovascular disease.

This study is subject to some limitations. For one, the sample size was relatively small relative to other MR studies, increasing the potential risk of type II error. The data used in this study are primarily based on Han Chinese patients, and there is no evidence of a causal relationship between LTL and CSVD in other ethnic populations, so the generalization of our findings to other ethnic groups may be limited.Further studies analyzing representative regional and ethnic populations will thus be necessary to validate these results, together with large-scale multicenter prospective analyses. Second, genetic variation is utilized as an instrumental variable in MR studies to satisfy the required assumptions. As the total elimination of potential confounding factors from MR methods remains challenging, however, it remains possible that the present results are subject to bias from pluripotency effects not identified at present. Many studies have shown that the construction of SNP-related PRSs can predict individualized disease risk, particularly in high-risk populations such as advanced age, hypertension, hyperglycemia, dyslipidemia, smoking, and high Hcy levels [26, 27]. However, other studies have shown that the PRS model is only slightly or not statistically significant compared to the traditional model [28]. The reason for the limited predictive power of the PRS may be related to genetic and environmental factors, as well as the multiple pathogenic mechanisms of the disease. In addition, poor predictive power may also be related to modeling algorithms. In the field of forecasting, the PRS model has also been studied by using machine algorithms, and good prediction efficiency has been achieved [29, 30]. Therefore, optimization algorithms and new machine algorithms can be considered in the future to improve the prediction ability of PRS models.

This study also included a relatively limited number of SNPs, reducing overall MR analysis accuracy such that weaker associations may have been missed. Further studies exploring the causal link between genetic predictors of LTL and CSVD risk that incorporate other LTL-related SNPs will thus be necessary to increase the overall power of these analyses and to further mitigate potential confounding variables. Large-scale analyses will also be vital to fully explore the association between LTL and other CSVD subtypes.

## Conclusion

In summary, based on analyses of multiple genetic models and MR methods using LTL-related SNPs as instrumental variables, these results suggest that TERT gene variants can contribute to an elevated risk of CSVD, and that shorter LTL may represent a causal factor for CSVD. Owing to the limited sample size in this study, however, additional MR studies utilizing GWAS or individual data will be essential to fully clarify the nature of this potential causal association.

## Data Availability

The data that support the findings of this study are available on request from the corresponding author. The data are not publicly available due to privacy or ethical restrictions.

## Abbreviations

TERT: Telomerase reverse transcriptase gene
LTL: Leukocyte telomere length
CSVD: Cerebral small vessel disease
SNPs: Single nucleotide polymorphisms
MR analysis: Mendelian randomization analysis
PRS: Polygenic risk score
IVs: Instrumental variables
SBP: Systolic blood pressure
LDL-C: Low-density lipoprotein cholesterol
Hcy: Homocysteine
95% CI: 95% Confidence interval
BMI: Body mass index
2SLS: Two-stage least square regression model
